# Items

**Published:** 1880-04

**Authors:** 


### Items.

COMMENCEMENT EXERCISES OF RUSH MEDICAL COLLEGE.

The thirty-seventh annual commencement exercises of Rush Medical College were held on Tuesday, February 24th, 1880, at 2:30 P. M., at the Third Presbyterian Church, corner of Ogden and Ashland avenues.

The following was the order of exercises:

1, Music; 2, Prayer; 3, Music; 4, Secretary's Report of the College Year; 5, Conferring of Degrees; 6, Music; 7, Charge of Acting-President Miller; 8, Response, on behalf of the class, by Dr. C. H. Lewis; 9, Music; 10. Valedictory Address, by Prof. Henry M. Lyman; 11, Music; 12, Benediction; 13, Music.

Following is the list of the Graduating Class of 1880:

*Officers of the Class.*—T. W. Brophy, President; S. S. McArthur, Vice-President; J. R. Robinson, Secretary; C. H. Lewis, Valedictorian.

*Executive Committee.*—E. H. Disbrow, S. C. Waters, H. Pritchard, E. S. Talbot, R. C. Meacher.

Johnson Armstrong, George Frank Allen, Winfield Ackley, M. D., Truman William Brophy, D. D. S., Samuel Cleland Bowman, John Anthony Badgley, Luther George Bass, B. A., Raymond Willis Battles, Franklin Albert Butterfield, George Frederick Bradley, Frank Preston Brown, Ignatius Bronish, Robert Leonard Boon, Fernando Wood Burdick, Marion Cazier, Willis Clay, David Gray Campbell, Charles Henry Carter, James Sylvester Collins, George Thomas Carpenter, D. D. S., John Francis Cully, Patrick William Conway, James Coolidge, John Franklin Cameron, Charles William Cruter, B. S., Francis Conrath, PH. G., James William Campbell, John Douglass Camerer, John Cicero Capps, James Calvin Clark, William George Dwyer, Joseph F.

Dicus, Ernest David Disbrow, Herbert William Davis, Calvin Todd Dripps, A. M., William Henry Earles, William Harter Everhard, Aaron Welch Edmiston, Henry Alonzo Ediston, William Henry Ellis, Hurford Edmur Farley, Francis William Fitzgerald, PH. G., Edward Fischer, A. B, Albert Lawrence Farr, George Henry Grose, John Colton Goodspeed, Charles Wesley Gordon, M. D., Joseph Godfrey, Myron Page Goodwin, William Greig, William Frank Graham, B. S., Levi Atchley Golden, William T. Green, William Elvis Harwood, William Arthur Hawley, Mortimer Lambert Hildreth, Herbert Dainy Hill, William Franklin Howe, Warner Holt, M. L., Samuel Ferdinand Hammond, Isaac William Hanna, Alvin Coles Hiester, William Clark Hill, Furman Springer Hand, Joseph Haven, J. Nathaniel Henry Huggins, John Alexander Inks, William Johnson, Claes William Johnson, Frank Joseph Jirka, Addison Coffea James, George Edwin Jackson, George Kernahan, Joshua Moffitt Kidney, Herbert Kendall, William Henry Lewis, William Bardwell Lyman, John Van Reed Lyman, Seth Ward Lacy, William Albert Lisman, Clinton Huntington Lewis, B. S., Peter Thames Mogstad, A. M., Nathaniel Massie McKitterick, George Bessore McCosh, Walter Scott Mason, Michael H. McGrath, Melvin Lazelle Moore, Julian Edwin Murray, Robert Edward Miller, John Hale Mannon, A. M., French Moore, Byron Coleman Meacher, Ezekial P. Murdock, A. M., Lucien Edward Murray, Willis Fremont Moore, Lewis Linn McArthur, John Leonard Mulfinger, PH. G., Marshall Thomas Martin, Joseph Converse McCormick, M. D., John Crittenden Nichols, John William Neill, Charles Frederick Nitz, Adamson Bentley Newkirk, Nelson Eugene Oliver, Daniel Samuel O'Brien, Harry Pritchard, Charles Nelson Palmer, Charles David Parks, Albert David Pyke, Francis Isaac Pinch, Eugene Beauharnoise Perry, James Franklin Paul. M. D., Will Aerious Quigley, John Hiram Quinn, Herman Reineking, George Jacob Rubleman, Monroe Griffith Reynolds, John A. Robinson, A. B., Emery Waland Roe, John Ritter, Charles Lester Stadler, Frank Buchanan Smith, Philip D. Shunk, Allen Vinton Smith, John Fritz Herbert Sugg, George Cassius Synon, B. S., E. Hudson Sammons, Thomas Jefferson Shaw, George Charles Somers, Walter Henderson Scott, Eugene

Colvin Southard, Albert John Scholl, Eugene Solomon Talbot, D. D. S., George Lytle Turner, William Wilson Torrence, Ely Judson Tanner, Milton Van Dyke, Walter Nathaniel Vilas, Charles Douglass Wright, Albert John Woodcock, Henry V. Worley, Peter N. Woods, M. D., Lisle Cummins Waters, Harmon Jackson Wall.

ALUMNI ASSOCIATION OF RUSH MEDICAL COLLEGE. GRAND PACIFIC HOTEL, FEBRUARY 24, 1880.

The Alumni Association of Rush Medical College was called to order at 8 p. m. by the President, Dr. Robert M. McArthur, of Ottawa, Ill. A large number of the old Alumni and the recent graduates were present.

The secretary's minutes of the last meeting were read and approved, and the resolutions offered by Dr. Ingals at the last annual meeting, regarding the course of training in the college, were adopted by the association.

"The Alumni of Rush Medical College desire to express, in a formal and public manner, the affectionate regard we entertain for our Alma Mater, our pride in her past history, and our confident hopes for her increased usefulness in the future. We hold that her supreme duty to the public, which has always accorded her a generous support, demands that her first aim and object should be to aid in exalting the attainments of the medical profession, and by so doing to increase its usefulness, power and respectability. As a mode of accomplishing these results we would respectfully offer to the board of trustees of the college the following suggestions:

"*First.* To increase the regular annual term of college instruction to a period of not less than nine months.

"*Second.* To require attendance on three full terms of medical lectures as a pre-requisite to admission to examination for the degree of Doctor of Medicine.

"*Third.* To institute preliminary examinations for students who apply for matriculation to the school, and admit only such as have at least a thorough English education."

The treasurer's report was next read and approved. The executive committee then presented the list of names nominated for officers of the association for the ensuing year. The officers were elected *viva voce*, the nominations of the committee being unanimously concurred in. The officers elected were as follows: President, Solon Marks, Milwaukee, Wis.; 1st Vice President, W. C. Hunt, Chicago; 2d Vice President, S. T. Ferguson, Minooka, Ill.; Secretary and Treasurer, E. Fletcher Ingals, Chicago; Executive Committee, James Nevins Hyde, T. Davis Fitch and F. Henrotin, Chicago.

The president's address was next in order. It will be found in the printed transactions. After the reading of the president's address the association adjourned to the upper rooms, where some time was spent in social converse before the guests were invited to the banquet hall. The Alumni were called by classes and were so seated that acquaintances were placed at the same table, an arrangement which greatly added to the pleasure of the evening.

The after-dinner speeches were as follows: Toasts—1. "The graduates of long ago," which was responded to by Dr. Solon Marks, of Milwaukee. 2. "Our visiting Alumni," responded to by Dr. Wm. Meachum, of Racine. 3. "The Spring Faculty," responded to by Dr. J. Suydam Knox, of Chicago. 4. "Clinical Instruction," responded to by Dr. S. D. Jacobson, of the Cook County Hospital. 5. "The best class graduated," responded to by Dr. C. T. Dripps. 6. "The Profession of Medicine," responded to by the well-known humorist Dr. Charles Gilman Smith. 7. The seventh toast, "Old Rush," was responded to by Prof. Delaskie Miller, who compared the growth of the college to that of our honored guest (Gov. Cullom), the chief executive of our State. He pointed out the steady and efficient growth which had taken place in the processes of medical education, and called attention to the vastly increased facilities which were now offered for thorough training in the healing art.

The master of ceremonies then called upon Dr. P. W. Brophy, a well-known dentist of this city, who, together with his fellows, Drs. Talbot and Carpenter, had that day, after great sacrifices for the last three years, added to their titles of D. D. S. that of Doctor of Medicine.

Dr. Brophy related the history of dentistry, and stated his firm conviction that dentistry must become one of the specialties of medicine before it could properly be called a profession. He believed that ere long it would be considered necessary for every dentist to have a thorough medical training before he was fitted for that branch of the profession.

The association then adjourned to meet on commencement day at the close of the next session.

A letter was then read from Prof. H. A. Johnson, of the Chicago Medical College, an alumnus of Rush College, Class of '52. Dr. Johnson had consented to reply to the sentiment in the 6th toast, but at the time of the gathering he was confined to his room by sickness. His letter was received with applause which was only limited by our regret that its author could not be present.

Many letters of regret had been received by the Secretary from the Alumni in distant parts of the country, but owing to the lateness of the hour only one of them could be read. At the request of the toast-master the secretary read the letter of Dr. Arthur Young, of Prescott, Wisconsin, class of 1853.

Dr. Young was one of those who knew the worth of a thorough medical education, and who had himself taken the full course of lectures before entering upon *his* professional career. He wrote feelingly of the departed ones who had lectured to the classes of his college days, and to many of his own classmates who "rested from their labors." With pride he pointed to the past and with firm hope to the future of Alma Mater.

THE CHICAGO MEDICAL COLLEGE.

The twenty-first commencement of the Chicago Medical College, Medical Department Northwestern University, occurred on March 30th in Plymouth Congregational church. A large audience was present, including members of the college with their friends. The music was furnished by a section of the Chicago orchestra. The exercises passed off pleasantly, many floral tributes being presented to the members of the graduating class.

The Rev. C. H. Everest offered the opening prayer. This was followed by the awarding of prizes. The standard prizes for the best graduating theses were awarded to F. H. Kimball and J. E. Bumstead. The prize offered by Dr. C. W. Earle, Dr. Smith's work on diseases of children, was awarded to N. E. Oliver for the highest proficiency in that department. A valuable work on surgery by S. D. Gross, offered by Dr. N. Senn, of Milwaukee, for the best anatomical preparation, was awarded to T. N. Miller. Prizes for the best work in anatomy were awarded to J. J. Fordyce and Frank Billings, in virtue of which they will be assistant acting demonstrators of anatomy. E. Andrews, Frank Johnson, and W. S. Gates also received honorable mention and will act as alternates to the above. Prof. R. L. Rea, the professor of anatomy, awarded to Frank Billings and A. M. Vail, a prize each for the best work in his department. The prize was a gold medal engraved with the letter "A" and a $10 gold piece. The prize for the best chemical work was awarded to Frank Billings, and for best labratory work by members of the junior class to E. L. Hollister; for best lecture notes to J. E. Marshall. These prizes consisted of standard works on medical chemistry.

Diplomas were then conferred to

THE GRADUATING CLASS

as follows:

Hiram H. Baldwin, James E. Bumstead, James Carter, J. Madison G. Carter, Andrew J. Coey, Charles E. Cook, John C. Cook, George R. Colby, William Dodd, A. A. Eddy, Wyman H. Fisher, William E. Gallup, Edgar A. Holmes, John I. Hostetter, Fred. O. Hoyt, Levi H. Johnson, Charles L. King, Frank H. Kimball, James J. Larkin, Frank H. Martin, Thomas N. Miller, George W. Mason, Nathaniel E. Oliver, Fred J. Parkhurst, Ernest Pierpoint, Lorenzo T. Potter, R. W. Emerson Puckett, John N. Pascoe, Fred. D. Rodgers, George U. Runcie, Willis S. Reynolds, Hein Terhorst, Homer H. Throop, Newton M. Wade, Thomas A. Ward, Milton P. White.

The ad eundem degree to graduates of other colleges was given to Benjamin F. Black and W. Frank Hicks. The appointments to the house staff of Mercy Hospital for the ensuing year were made to James J. Larkin, Frank H. Martin, and L. T. Potter, all of them being members of the graduating class. These degrees and appointments were accompanied by a few appropriate words by Prof. N. S. Davis.

A feeling response on behalf of the class was made by George W. Mason, and the valedictory address on behalf of the faculty by Prof. E. W. Jenks.

At the close of the exercises it was announced that the practitioners' course of study to last four weeks would commence at once.

### A BUSINESS MEETING.

In the evening the annual business and fourteenth reunion of the Alumni Association was held at the Tremont House. The business meeting was presided over by Dr. Norman Bridge, first vice-president, who, on assuming the chair, referred in a few appropriate words to the death of Dr. John M. Woodworth, president of the association.

During the absence of the committee appointed to nominate officers for the ensuing year, the necrologist's report was read, and showed a record of four deaths in the roll of membership during the past year. These were Dr. D. H. Alvis, class of '70, Kewaunee, Ill.; Dr. Zenophan Chapman, class of '74, Leadville; Dr. F. A. Beck, class of '77, Chicago, and Dr. G. H. A. Sienank, class of '77, Silver Lake, Kan. On the the close of the reading of the necrologist's report, Dr. Norman Bridge again referred to the death of President Woodworth, paying an eloquent tribute to the memory of the deceased gentleman. Other members of the association also referred to the deceased president and members.

The nominating committee then reported the following list of officers, which was unanimously adopted:

Dr. W. E. Quine, president, Chicago; Dr. Webber, first vice president, Detroit; Dr. Fitch, second vice-president, Rockford; Dr. D. A. K. Steele, secretary and treasurer, Chicago; Dr. Waxham, necrologist, Chicago.

Dr. Steele, who has served as secretary and treasurer of the association for over five years, gave notice of his intended resignation at the next annual reunion.

### THE BANQUET.

This closed the business meeting and the members of the association to the number of one hundred, accompanied by a dozen or so members of the present year's graduating class, repaired to the ladies' ordinary, where the social exercises of the evening were indulged in. Dr. Quine, the newly-elected president, occupied the seat of honor, upon assuming which he fittingly thanked the association for the honors conferred upon him.

The programme had assigned Dr. Lyman Ware to deliver the address of welcome, but owing to the absence of that gentleman the banquet was proceeded with in an informal way. At its close toasts and responses followed in rapid succession. "The Old Folks" was responded to by Prof. R. L. Rea, of this city; "Big Brothers," by Dr. Webber, of Detroit; "The Baby," by Dr. F. H. Kimball, of Chicago, and "First Cousins," by Hon. J. S. Grinnell, of Iowa. A number of impromptu toasts were given and responded to by Drs. Hosmer A. Johnson, Rev. Dr. Edwards and others, the gathering indulging in the pleasures of the occasion until a late hour.

### Woman's Medical College of Chicago.

The tenth annual commencement of the Woman's Medical College of Chicago was held at the Union Park Congregational Church, March 2d, 1880.

Prof. Wm. H. Byford, president, conferred the degree upon Addie Akins, Jennie M. Dobson, A. M. Davenport, Jennie E. Hayner, Elizabeth McKittrick, Emma M. Nichols, Julia M. Patten, Amelia Augur Platt, Mary Lyman Rockwell and Eugenia A. Watts.

A special certificate was given Mrs. M. L. Rockwell, Mrs. Amelia A. Platt and Mis A. M. Davenport for general proficiency and for attendance upon three full courses of lectures.

Prizes for special merit, as evinced on examinations, were awarded to Miss Jennie E. Hayner and Miss A. M. Davenport.

The class valedictory was delivered by Miss Julia M. Patten.

The faculty address by Prof. I. N. Danforth.

An elegant entertainment was given by Prof. C. W. Earle, at his residence, at the close of the exercises, to the graduating class, the alumni of the college and their friends.

Miss Emma M. Nichols, M.D., has been appointed House Physician at the Woman's Hospital of the State of Illinois, and Miss

Jennie E. Hayner, M.D., Resident Physician at the Chicago Hospital for Women and Children. The appointments were made after a competitive examination.

MEDICAL DEPARTMENT, IOWA STATE UNIVERSITY.

The commencement of the Medical Department of the Iowa State University was held in the opera house at Iowa City, on Wednesday evening March 3d.

President Pickard conferred the degree of M.D. upon the following ladies and gentlemen:

Miss H. A. Conniff, Miss Olive Morris, L. H. Munn, W. J. Saunders, T. J. Shuell, R. J. C. Dodds, A. C. Jennis, G. M. Barber, John Riley, P. O'Hair, J. C. Davies, F. S. Johnson, W. K. Williams, H. Parrish, C. E. Nichols, W. E. Edgerton, J. C. Wright, John R. Coxe, C. P. Dolan, T. J. Williams, A. D. Paine, L. L. Renshaw.

This class had been examined for two days by a board of examiners, members of the Iowa State Medical Society, consisting of Dr. Fitch, Charles City, chairman; Dr. Clark, Grinnell; Dr. Dean, Muscatine; Dr. Wright, Carroll; Dr. Kersey, Stuart; Dr. Thomas Walnut, secretary, who together with the faculty, passed upon the qualifications of students.

T. S. Johnson delivered the valedictory for the class, and Dr. W. F. Peck, dean, delivered the address for the faculty.

THE Alumni Association of Cook County Hospital Internes held its annual reunion at the Tremont House, February 23d. The association now contains more than forty names on its membership roll, though not more than half that number were present. One or two members of the medical board were also present. Dr. Fox of Milwaukee, the president, was unavoidably detained at the eleventh hour, and Dr. Strong was made chairman for the evening. The usual routine business was transacted, letters of regret from absentees read, and Dr. Gephart of Kansas elected president for the ensuing year. A bountiful repast was served in the ladies' ordinary, and the alumni finally adjourned amid a cloud of smoke at a late hour.

ROSWELL PARK, *Permanent Secretary.*

AMERICAN MEDICAL ASSOCIATION.

PHILADELPHIA, 1400 PINE STREET,
*Southwest corner Broad.*

The thirty first annual session will be held in the city of New York, on Tuesday, Wednesday, Thursday and Friday, June 1, 2, 3 and 4, 1880, commencing on Tuesday at 11 a. m.

"The delegates shall receive their appointment from permanently organized State Medical Societies, and such County and District Medical Societies as are recognized by *representation in their respective State Societies*, and from the Medical Department of the Army and Navy of the United States."

"Each State, county and district medical society entitled to representation shall have the privilege of sending to the association one delegate for every ten of its regular resident members, and one for every additional fraction of more than half that number; *provided*, however, that the number of delegates for any particular State, territory, county, city, or town shall not exceed the ratio of one in ten of the resident physicians who may have signed the code of ethics of the association."

Secretaries of medical societies as above designated are earnestly requested to forward, *at once*, lists of their delegates.

SECTIONS.—"The chairman of the several Sections shall prepare and read in the general sessions of the association, papers on the advances and discoveries of the past year in the branches of science included in their respective Sections. * * *"—BY-LAWS, Art. II. Sec. 4.

*Practice of Medicine, Materia Medica and Physiology.*—Dr. J. S. Lynch, Baltimore, Md., *Chairman;* Dr. W. C. Glasgow, St. Louis, Mo., *Secretary.*

*Obstetrics and Diseases of Women and Children.*—Dr. Albert H. Smith, Philadelphia, Pa., *Chairman;* Dr. Robert Battey, Rome, Ga., *Secretary.*

*Surgery and Anatomy.*—Dr. W. T. Briggs, Nashville, Tenn., *Chairman;* Dr. C. Powell Adams, Hastings, Minn., *Secretary.*

*Medical Jurisprudence, Chemistry, Pyschology, State Medicine and Public Hygiene.*—Dr. Jas. F. Hibberd, Richmond, Ind., *Chairman;* Dr. Thos. F. Wood, Wilmington, N. C., *Secretary.*

*Ophthalmology, Otology, and Laryngology.*—Dr. Bolling A. Pope, New Orleans, La., *Chairman;* Dr. Eugene Smith, Detroit, Mich., *Secretary.*

The following committees are expected to report:

*On Prize Essays.*—Dr. Austin Flint, New York, *Chairman.*

*On Necrology.*—Dr. J. M. Toner, Washington, D. C., *Chairman.*

*On Catalogue of National Library.*—Dr. H. C. Wood, Pa., *Chairman.*

*On Ozone.*—Dr. N. S. Davis, Chicago, Ill., *Chairman.*

*On Metric System.*—Dr. T. Parvin, Indianapolis, Ind., *Chairman.*

*On State Medical Societies.*—Dr. S. D. Gross, Philadelphia, *Chairman.*

To be acted upon:

*Changes in By-Laws* proposed by Committee on the Address of the President.

1. Expunge from Section III everything relating to Prize Essays, and the Committee on Prize Essays.

2. Introduce into Section II the following laws:

*Prize Essays.*

(*a.*) There shall be four annual prizes of two hundred and fifty dollars each, which shall be awarded at the close of the second year after announcement, as hereinafter explained, for strictly original contributions to medical and surgical progress.

(*b.*) It shall be the duty of the chairman of each of the following four Sections: 1. Practical Medicine, Materia Medica, and Physiology; 2. Obstetrics and Diseases of Women and Children; 3. Surgery and Anatomy; 4. State Medicine and Public Hygiene, to appoint annually before the adjournment of the meeting of the Association three members of ability and good judgment, who shall constitute a Committee of Selection, and who shall, within thirty days thereafter, select and publicly announce for competitive investigation and report, a subject belonging to one or other of the branches of medicine included in the title of the Section.

(*c.*) It shall also be the duty of the chairman of each of the Sections mentioned to appoint annually a Committee of Award, consisting of three experts, who shall carefully examine the essays offered for competition, and if any one shall be found worthy of the prize as a substantial contribution to medical knowledge, to recommend the same to the Association.

(*d.*) All essays placed by their authors for competition shall be in the hands of the chairman of the respective Committees of Award on or before the first day of January preceding the meeting of the Association at which the reports of the committees are required to be made.

(*e.*) All Prize Essays shall be considered as the property of the Association.

(*f.*) The names of the authors of the competing essays shall be kept secret from the committees by such means as the latter may provide.

(*g.*) Membership in either of the two committees shall not debar from membership in the other; nor shall membership in the Committee of Selection exclude a member from the privilege of offering a competing essay.

*Amendments proposed by* DR. JOHN H. RAUCH.

AMENDMENT TO THE CONSTITUTION.

Article II., second paragraph, after "Army and Navy" insert "and the Marine Hospital Service of the United States."

Article II., fourth paragraph, at the end insert "the Marine Hospital Service of the United States shall be entitled to one delegate."

Will you kindly send to the undersigned a list of your members, with their residences, in order that a correct record may be made of all who are in affiliation with this body?

W. B. ATKINSON,
*Permanent Secretary.*

ILLINOIS STATE MEDICAL SOCIETY.—The next regular annual meeting of this Society will be held in Belleville, Ill., commencing on the morning of the *third Tuesday* in May. The local Committee of Arrangements are making all needed arrangements for a good meeting. The following committees are expected to report:

*Committee of Arrangements.*—Drs. T. B. Moore, Belleville; Washington West, Belleville; T. M. Armstrong, Madison Co.; Wm. R. McKenzie, Randolph Co.; H. Z. Gill, Jerseyville.

*Committee on Practical Medicine.*—W. S. Caldwell, Warren; F. B. Haller, Vandalia; J. S. Whitmire, Metamora.

*Committee on Surgery.*—W. Hill, Bloomington; A. C. Rankin, Loda; J. G. Harvey, Grove City.

*Committee on Obstetrics.*—G. W. Nesbitt, Sycamore; E. L. Herriott, Grafton; I. W. Fink, Hillsboro.

*Committee on Gynæcology.*—T. Davis Fitch, Chicago; J. Y. Campbell, Paxton; L. H. Corr, Carlinville.

*Committee on Ophthalmology.*—W. T. Montgomery, Chicago; J. M. Everett, Dixon; J. P. Johnson, Peoria.

*Committee on Drugs and Medicines.*—C. B. Johnson, Champaign; Jehu Little, Bloomington; Thos. Whitten, Irving.

*Committee on Necrology.*—T. F. Worrell, Bloomington; G. W. Albin, Neoga; E. Ingals, Chicago.

*Vacancies in Judicial Council.*—F. B. Haller, Vandalia; E. Ingals, Chicago; M. W. Walton, Ridott.

*Committee on State Register.*—E. P. Cook, Mendota; F. L. Matthews, Springfield; D. S. Booth, Sparta.

SPECIAL COMMITTEES.

*Committee on Croup.*—H. Z. Gill, Jerseyville.

*Committee on Diseases of Children.*—Chas. W. Earle, Chicago; M. W. Walton, Ridott.

*Committee on Pelvic Cellulitis (Parametritis).*—P. L. Dieffenbacher, Havana.

*Committee on Pneumonia.*—J. Maclay Armstrong.

*Committee on Antiseptic Surgery.*—Edwin Powell, Chicago.

*Committee on Abnormal Thermal Conditions in Diseases, and the Means of Controlling Them.*—J. H. Hollister, Chicago.

APROPOS of the interesting paper on monsters, which appears in another column, it is reported in a medical publication foreign to this city, that a new institution is to be established here, to be called the Union College of Medicine, with a faculty to be composed of representatives of all the 'pathies! Monstrum horrendum!

